# Treatment Options and Antifungal Resistance in Pediatric *Candidozyma auris* (Previously *Candida auris*) Infections: A Systematic Review of Clinical Outcomes

**DOI:** 10.3390/microorganisms14010228

**Published:** 2026-01-19

**Authors:** Konstantinos Stergiou, Kanellos Skourtsidis, Georgios Kiosis, Despoina Ioannou, Vasilis-Spyridon Tseriotis, Vasileios Fouskas, Sofia Karachrysafi, Elias Iosifidis, Emmanuel Roilides, Theodora Papamitsou, Maria Kourti

**Affiliations:** 1 Research Team “Histologistas”, Interinstitutional Postgraduate Program “Health and Environmental Factors”, School of Medicine, Faculty of Health Sciences, Aristotle University of Thessaloniki, 54124 Thessaloniki, Greece; konstantinos.d.stergiou@outlook.com (K.S.); kskour@auth.gr (K.S.); gkiosis@auth.gr (G.K.); dioana@auth.gr (D.I.); fvasillis79@gmail.com (V.F.); sofia_karachrysafi@outlook.com (S.K.); thpapami@auth.gr (T.P.); makourti@auth.gr (M.K.); 2 Laboratory of Histology-Embryology, School of Medicine, Faculty of Health Sciences, Aristotle University of Thessaloniki, 54124 Thessaloniki, Greece; 3 Department of Neurology, Agios Pavlos General Hospital of Thessaloniki, Leoforos Ethnikis Antistaseos 161, Kalamaria, 55134 Thessaloniki, Greece; vasilistseriotis@hotmail.com; 4 Laboratory of Clinical Pharmacology, School of Medicine, Faculty of Health Sciences, Aristotle University of Thessaloniki, 54124 Thessaloniki, Greece; 5 Third Department of Pediatrics, School of Medicine, Faculty of Health Sciences, Aristotle University of Thessaloniki, Hippokration General Hospital, 54642 Thessaloniki, Greece; iosifidish@auth.gr; 6 Pediatric & Adolescent Hematology Oncology Unit, 2nd Pediatric Department, Faculty of Health Sciences, Aristotle University of Thessaloniki, AHEPA Hospital, 54124 Thessaloniki, Greece

**Keywords:** *Candida auris*, *Candidozyma auris*, children, neonates, antifungal resistance, combination therapy, nosocomial infections, multidrug-resistant fungi, invasive fungal infection, treatment outcomes

## Abstract

*Candidozyma auris* (previously named *Candida auris*) has been recognized as a significant public health threat due to its extensive transmission in hospital settings, high mortality rates, and multidrug resistance. Evidence regarding optimal antifungal treatment in children remains limited. The present systematic review aims to synthesize available evidence on pediatric *C. auris* infections, focusing on antifungal treatment, resistance profiles, and clinical outcomes. A systematic search was conducted across PubMed, Scopus, and Web of Science, identifying case reports and case series of pediatric patients with confirmed *C. auris* infection. Data were extracted on demographics, comorbidities, infection site, antifungal therapy, and outcomes. Risk of bias was assessed using JBI Critical Appraisal checklists. Fourteen studies comprising 62 patients were included, with most cases being bloodstream infections. *C. auris* showed widespread fluconazole resistance and variable susceptibility to amphotericin B. Echinocandins were the most commonly used agents, generally associated with survival. Overall mortality was 35%, similar to that reported for adults. Combination therapy showed numerically higher survival, although given the small sample size and heterogeneity of treatment regimens, no comparative inferences can be made. Pediatric *C. auris* infections mirror adult patterns of antifungal resistance and mortality. Echinocandins remain first line therapy; however, the emergence of echinocandin resistance underscores the urgent need for antifungal stewardship, standardized pediatric guidelines, and novel antifungal development.

## 1. Introduction

Fungal infections constitute a major global cause or morbidity and mortality, especially among immunocompromised individuals and hospitalized patients with severe comorbidities and chronic conditions. The epidemiology of fungal infections in recent years saw the introduction of novel pathogens, expansion of the geographic distribution of established ones, and the highly alarming problem of antifungal resistance [1]. Increasing resistance to antifungal agents among pathogenic fungi remains a longstanding but under-recognized challenge, especially considering the mainstream attention drawn to antibiotics resistance in recent years. Wide azole compound use, not only as human and veterinary medicine but also in agricultural or industrial settings, has applied evolutionary pressure, promoting the emergence of environmental fungal strains with acquired resistance to this broad class of drugs [2]. Among these pathogens, *Candidozyma auris*, a multidrug resistant yeast, has become a global healthcare concern of growing significance for the scientific community. *Candidozyma auris* (previously named *Candida auris*) was first identified in 2009, isolated from the external ear canal of a patient in Japan [3]. Fifteen years since, this newly recognized species of yeast has disseminated across five continents, claiming lives and causing numerous nosocomial outbreaks [4]. *C. auris* is remarkable due to its ability to persist on skin and inanimate surfaces, making elimination and infection control a challenge. Nearly all isolates exhibit in vitro resistance to fluconazole, approximately one-third to amphotericin B, and about 6% to echinocandins, with sporadic yet truly alarming reports of pan-resistance—strains resistant to all three major antifungal families [5]. Phenotypic and genotypic studies of isolates from different regions have identified several distinct geographic clades, with a particular set of virulent factors and susceptibility profiles [6]. In addition to the adhesive properties of the yeast, binding to a broad range of biotic and abiotic surfaces, *C. auris* displays the notable ability to form biofilms. Fungal cells are dispersed in an extracellular matrix, fortifying against antifungals and the host’s immune mechanisms, while also facilitating efficient colonization of skin and surfaces [7]. A biofilm-forming pathogen is equipped with unique components, such as mannan–glucan complexes, hindering penetration of antifungals and more active drug-efflux pumps, expelling drugs more efficiently. Essential for this mode of growth is a dominant adhesin, Surface Colonization Factor 1 (SFC1), in combination with a complementary adhesin, IFF4109 [8]. The initial adhesion is followed by cell proliferation and cluster formation. These clusters merge and mature into an organized form, displaying multiple layers of cells, and channels for nutrient absorption and waste removal.

Timely and accurate *C. auris* detection is crucial for preventing further dissemination of the pathogen. However, diagnostic and therapeutic methods have not evolved at the same pace as the rapid emergence of new fungal pathogens, leading to frequent misidentification and unfavorable outcomes due to suboptimal antifungal therapy [9]. *Candidozyma auris* colonies grow pink or white, while demonstrating high heat tolerance, with growth at high temperatures (40–42 °C), characteristics that overlap with other yeasts. Conventional biochemical and phenotypic assays, often misclassify *C. auris* as other species, most commonly *Candidozyma haemuli* or *Debaryomyces hansenii*, resulting in inappropriate antifungal selection and worse patient outcomes. More robust diagnostic approaches include sequencing of the internal transcribed spacer (ITS) region and matrix-assisted laser desorption ionization time-of-flight mass spectrometry (MALDI-TOF MS) [10]. Prompt identification is essential, given the organism’s reduced susceptibility to common disinfectants and antifungals, and the importance of early treatment for more favorable patient outcomes. Critically ill and immunocompromised patients are heavily susceptible to colonization, with person-to-person transmission and transmission via surfaces frequently causing extensive nosocomial outbreaks. Indwelling devices, such as central venous catheters (CVCs) and urinary catheters, are often colonized and may serve as portals for bloodstream infection, with very high mortality rates [11]. Major risk factors for invasive candidiasis include intensive care unit admission, malignancy, concurrent bacterial infections, exposure to broad-spectrum antibiotics or antifungals, immunosuppressive therapy, and extremes of age [4]. Children and neonates are a particularly vulnerable population, especially bearing in mind immature immunity and extremely premature neonates. Evidence regarding optimal antifungal treatment of pediatric *C. auris* patients remains limited, with the correlation between in vitro susceptibility and clinical outcomes not fully understood [12]. Despite the growing global concern surrounding antifungal resistance, comprehensive evidence guiding pediatric pharmacotherapy and management of resistance remains scarce and fragmented across case reports. The present review aims to systematically consolidate available pediatric cases of *C. auris* infection to better characterize treatment strategies, resistance characteristics, and clinical outcomes associated with the above treatments. Given the shallow pool of reported cases and the possibility of publication bias, the goal is not to establish definitive treatment recommendations but to provide a structured overview that highlights current therapeutic knowledge and identifies critical gaps for future research.

## 2. Materials and Methods

### 2.1. Protocol and Registration

The present systematic review was conducted following the recommendations of the PRISMA statement and PRSMA checklist is shown in Supplementary Table S1 [13]. The review protocol was registered on the Open Science Framework (OSF) DOI: 10.17605/OSF.IO/F5KBZ, with a PROSPERO record created in September 2025. Any deviations from the protocol would be reported in full transparency in the final manuscript.

### 2.2. Research Question and Objectives

The primary search objective was to identify antifungal agents (echinocandins, azoles, amphotericin B, or others) used in pediatric patients with *C. auris* infection to determine the reported clinical outcomes associated with the above treatments (e.g., mortality, success/failure of treatment, recurrence) and finally, to observe and document the patterns of antifungal resistance, if any, that occur. The secondary research objective was to determine how treatment approaches (monotherapy vs. combination therapy, different antifungal classes, effectiveness of prophylaxis) relate to clinical outcomes and resistance development.

### 2.3. Eligibility Criteria

Studies were selected based on a predefined PICO framework, as described in Table 1. Included studies were those with cases of confirmed *C. auris* infection in pediatric patients aged 0–18 years, receiving antifungal treatment (echinocandins, azoles, amphotericin B, novel antifungals). Eligible study designs included both randomized and non-randomized studies, observational studies, case series, and individual case reports. Articles labeled as “reviews” were included only when new primary clinical data were present (e.g., original patient cases) in addition to summary material. Studies with only adult populations, without pediatric subgroup data, non-human studies (in vitro/animal), and studies (or pediatric cases within those studies) not reporting any antifungal treatment or clinical outcome were excluded. Pure narrative or systematic reviews without primary data were also excluded.

### 2.4. Search Strategy

Three major databases were searched (PubMed, Scopus, Web of Science) for articles published from 2009 through 18 October 2025. The search strategy was designed to maximize sensitivity without sacrificing clinical relevance to the review’s objective. Controlled vocabulary (MeSH) was combined with free-text items to provide strong coverage of synonyms. Google Scholar was also used for exploratory purposes. A search strategy example can be found in Supplementary Materials, Table S2. Finally, techniques such as forward and backward citation searching were deployed.

### 2.5. Screening and Data Management

All retrieved records were exported into a single reference management file. Duplicates were automatically removed using the online tool “Catchii”, with manual verification following to ensure accuracy [14]. Subsequently, two independent reviewers screened titles and abstracts against the inclusion and exclusion criteria. The second stage of screening included full text, with both reviewers blinded to each other’s decisions. Disagreements were resolved through discussion and consensus. Finally, eligible studies were imported to the reference manager “Mendeley”.

### 2.6. Data Extraction

Data extraction was performed by two independent investigators in two stages. The first stage was primary data extraction, where the following entities were extracted: Article metadata (authors, year of publication, country, study design, and DOI), patient demographics (age, sex, comorbidities, immune status), clinical setting, infection site, method of *Candida auris* identification, reported antifungal susceptibility, antifungal agent or agents used (drug name, dose, duration), timing of treatment initiation, adjunctive management of the infection, clinical outcome, development of resistance during treatment, length of stay, and adverse effects related to antifungals. Attributable mortality (i.e., deaths directly caused by *Candidozyma auris* infection) and time-defined mortality endpoints were not prespecified outcomes, as these parameters were inconsistently reported or absent across the included case reports and case series. The second stage was risk of bias assessment, based on the Joanna Briggs Institute (JBI) Critical Appraisal Checklists for Case Reports or Case Series, selecting the version appropriate for each study design [15]. Quality ratings were not used to exclude studies, given the limited data available, but to inform the interpretation of findings and highlight areas of greater or lesser confidence during narrative synthesis.

## 3. Results

### 3.1. Study Selection and PRISMA Flow Diagram

**Figure 1:**
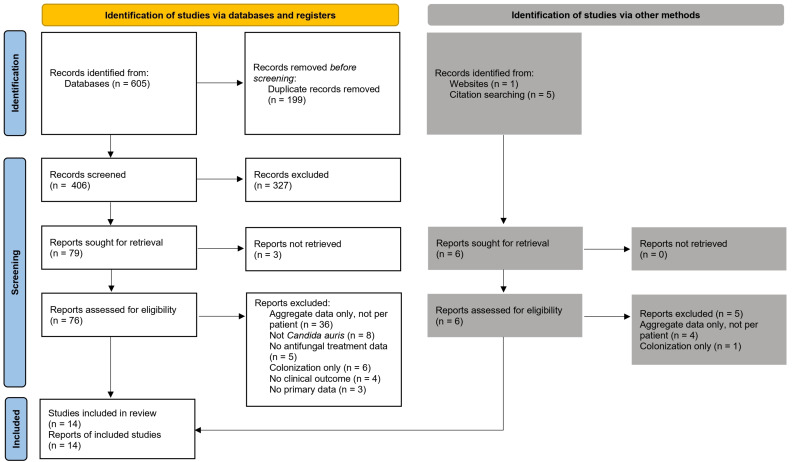
PRISMA flow diagram.

A total of 605 records were identified through database searches (PubMed n = 127, Scopus n = 320, Web of Science n = 158) and six records through other sources. Following the removal of 199 duplicates, 406 records were screened by title and abstract. The study selection process is illustrated in the PRISMA 2020 flow diagram in Figure 1 and the PRISMA checklist is shown in Supplementary Materials, Table S1.

The included studies comprised six case reports, six case series, one clinical note, and one outbreak report, published between 2011 and 2025. Most cases originated from Asia (n = 10), followed by Europe (n = 2) and South America (n = 2). The patient population included neonates (full-term, preterm, and extremely preterm), infants, children, and adolescents, totaling 62 patients, with a median age of 20.5 days and an IQR of 718 days. Common comorbidities included preterm, sepsis, congenital anomalies, respiratory distress syndrome, and necrotizing enterocolitis. The most common clinical manifestation of *C. auris* was bloodstream infection (n = 54), followed by urinary tract infection (n = 10) and less frequent sites such as cerebrospinal fluid, external or middle ear, and purulent secretions.

### 3.2. Overview of Included Studies

Ozen et al. reported two pediatric patients with extensive skin and inhalation burns, both of whom had received empirical fluconazole and broad-spectrum antibiotics prior to the identification of *C. auris*. In both cases, candidemia was associated with central venous catheter (CVC) use. Adjunctive management included surgical wound care (escharotomy) and CVC removal. Patient 1 was treated with caspofungin, whereas Patient 2 received combination therapy with liposomal amphotericin B and voriconazole. Antifungal therapy continued for over 40 days, after which both patients achieved negative blood cultures [16].

Das et al. described a case of a 13-year-old male, referred to the emergency department for failure to wean off ventilatory support over one week, after being revived from a cardio-respiratory arrest. The patient received broad spectrum antibiotics and fluconazole. The patient was admitted to the intensive care unit (ICU), where blood cultures grew yeast, initially misidentified as *Debaryomyces hansenii*. Eventually the patient responded to treatment and was discharged. *Candidozyma auris* was retrospectively identified by ITS sequencing [17].

Shin et al. reported a 9-year-old male with recurrent otitis externa following tympanoplasty. Culture of the otorrhea revealed *C. auris* among other pathogens, and the patient received micafungin. Subsequently, he was admitted for a third tympanoplasty with mastoidectomy while continuing to receive micafungin and antibiotic therapy. Fifteen days postoperatively, a culture of the otorrhea confirmed the clearance of *C. auris* [18].

Abastabar et al. presented a 14-year-old female with external otitis unresponsive to empirical antibiotics and topical corticosteroids. White to cream discharge was collected for culture identifying *C. auris* by ITS sequencing and MALDI-TOF MS. Antifungal treatment of topical nystatin and oral terbinafine resulted in no clinical improvement, and no follow-up was reported [19].

Anirima et al. summarized the efforts towards the investigation and containment of an outbreak in a pediatric intensive care unit (PICU), involving four patients with suspected sepsis, in whom blood cultures yielded multidrug resistant *C. auris*. All isolates were resistant to fluconazole, with only one isolate susceptible to amphotericin B. The affected patients had severe comorbidities, including an extremely preterm neonate, all received antifungal therapy. One patient survived, two died, and one was self-discharged against medical advice [20].

Lee et al. described three cases of *C. auris* fungemia, two of which involved pediatric patients [21].

Patient 1 was a one-year-old female who underwent emergency surgery for removal of foreign bodies obstructing the respiratory tract. Postoperatively, she received antibiotics and had a central venous catheter (CVC) inserted. Blood cultures initially yielded *Candida albicans*, and amphotericin B deoxycholate was administered. The treatment was later switched to fluconazole, during which time *C. auris* was isolated. Both fungi persisted until CVC removal, after which blood cultures returned negative and the patient was discharged.

Patient 2, who had hemophagocytic lymphohistiocytosis and a history of colectomy, was transferred for further chemotherapy. Following the onset of fever, blood cultures grew *C. auris*. Fluconazole therapy was initiated, and a peripheral vascular catheter was removed; however, the patient ultimately died from septic shock due to persistent fungemia.

Calvo et al. reported a hospital outbreak involving 18 critically ill patients, 13 of whom were children or neonates aged between 2 days and 14 years. All patients had received antibiotics prior to *C. auris* diagnosis and were managed with CVC removal and antifungal therapy. Susceptibility testing revealed sensitivity to anidulafungin and, in 50% of the cases, to amphotericin B. Among the thirteen pediatric patients, nine survived [22].

Chowdhary et al. presented a series of *C. auris* fungemia cases comprising twelve isolates, of which five corresponded to pediatric patients. One case was excluded from the present analysis as no antifungal therapy had been administered. All four patients included had severe comorbidities. All isolates were resistant to fluconazole, whereas other azoles showed potent activity. One of the four patients died [23].

Chandramathi et al. described 17 neonatal cases of *C. auris* sepsis. All patients presented with comorbidities such as respiratory distress syndrome (RDS) or congenital anomalies and had received antibiotics. Fluconazole prophylaxis was routinely used in extremely low birth weight infants within the unit. All isolates were sensitive to voriconazole and micafungin, resistant to fluconazole, and showed intermediate susceptibility to amphotericin B. Ten of the seventeen infants survived; only one of those treated with micafungin died [24].

Ramya et al. characterized five low birth weight, extremely premature neonates with late-onset *C. auris* sepsis, indicating nosocomial transmission. All isolates were susceptible to micafungin, which was administered to all patients, with two also receiving voriconazole. Only one neonate survived [25].

Alvarado et al. reported eight cases of invasive infection due to *C. auris* and *C. haemuli* among neonates and infants. All isolates demonstrated resistance to amphotericin B but susceptibility to caspofungin. Seven of eight patients received caspofungin, and three received combination therapy. All three patients treated with combination regimens achieved microbiological cure, one of whom had a prolonged hospital stay due to her cardiopulmonary condition and subsequently died [26].

Mirhendi et al. reported a 30-month-old male diagnosed with necrotizing pneumonia who underwent partial lobectomy for treatment of a pneumatocele. Neurological deterioration prompted transfer to the pediatric intensive care unit (PICU), where computed tomography (CT) revealed acute hydrocephalus. Following stabilization, serial lumbar punctures (LPs) demonstrated budding yeast cells, subsequently identified as *C. auris*. The patient initially received fluconazole, followed by liposomal amphotericin B combined with oral flucytosine, resulting in gradual clinical improvement. The patient was later discharged against medical advice, and no follow-up data were available [27].

Erkol et al. described a 17-year-old male treated for autoimmune encephalitis in the PICU. Urine cultures yielded *C. auris*, identified by MALDI-TOF MS as resistant to fluconazole and susceptible to echinocandins and amphotericin B. Ultrasonography revealed a fungal bezoar within the bladder, which was surgically excised. Caspofungin therapy was initiated; however, four days later, *C. auris* was also isolated from blood cultures. In light of emerging caspofungin resistance, therapy was switched to liposomal amphotericin B. After a total hospital stay of six months, the patient was discharged [28].

Chen et al. reported two cases of preterm infants with *C. auris* fungemia admitted to a neonatal intensive care unit (NICU).

Patient 1 was a two-month-old male whose admission cultures were positive for *C. auris*, suggesting infection prior to hospitalization. He was treated initially with fluconazole, without improvement and subsequently itraconazole and was discharged after seven days with clearance of fungemia.

Patient 2 was a one-day-old neonate of low birth weight admitted to the same unit. The same antifungal regimen (fluconazole followed by itraconazole) was administered, and the patient achieved microbiological clearance prior to discharge [29].

### 3.3. Antifungal Treatment

Azoles were the most frequently used class of antifungal agents (66%), followed by echinocandins (58%) and amphotericin B formulations (39%), while other antifungal agents accounted for 3% of treatments. Information on the timing of antifungal administration was unavailable for 17 patients, due to insufficient reporting of treatment timelines. Out of 27 patients receiving empirical treatment, 25 received fluconazole. Early treatment decisions were often influenced by misidentification of *C. auris.* Targeted pharmacotherapy predominantly included echinocandins or amphotericin B formulations. Treatment initiated after *C. auris* confirmation or susceptibility testing was categorized as targeted therapy. Out of 41 patients receiving targeted therapy, 26 received echinocandins and 17 amphotericin B. Timing information and outcomes are summarized in Table 2.

Combination therapy was employed in 10% of cases (n = 6), with five patients surviving and one dying. Notably, for one survivor of the combination therapy group with external ear infection, no clinical improvement was reported, whereas one patient who died had achieved microbiological clearance but succumbed to cardiorespiratory deterioration [19,26]. Monotherapy was administered in 40.3% of the cases (n = 25), of whom twenty-one survived and nine died; however, three of these patients were discharged against medical advice, introducing uncertainty in outcome assessment [20,26,27]. Five patients (8%) received sequential monotherapies, with four of them surviving (80%). Additionally, 39% of the patients (n = 26) received more than one antifungal agent during the course of treatment, though it was not possible to distinguish whether these regimens represented true combination therapy or sequential monotherapies. Fourteen patients out of the above group survived (53.8%). The above data are summarized in Table 3.

### 3.4. Clinical Outcomes

Overall, 40 patients survived and 22 died, with a survival rate of 64.5%. This proportion aligns broadly with previously reported mortality rates for *C. auris* infections. Among the twenty-two reported deaths, attributable mortality was explicitly assessed in only two cases, one death being directly attributed to sepsis caused by *C. auris* infection [21], while one was reported as non-attributable, occurring as a result of cardiopulmonary deterioration [26]. In the remaining 20 cases, no safe conclusion could be drawn on whether death was infection related. Time-defined mortality was reported inconsistently as well. Thirty-day survival status was available for only seven patients, of whom three survived beyond thirty days and four died within thirty days. For the remaining 15 fatal cases, the timing of death relative to infection onset or definitive *C. auris* diagnosis was not specified.

Immune status was also not consistently reported across studies and was therefore determined through available clinical information, where possible. Patients were classified as “immunocompromised” when a medication (e.g., corticosteroid or chemotherapy) or clinical history indicated immune impairment. In cases where no such factors were described, patients were considered “immunocompetent”. When insufficient information was available to support classification, immune status was recorded as “unclear”. Among immunocompetent individuals (n = 13), 11 survived and 2 died, while among immunocompromised patients (n = 40), 25 survived and 15 died. Survival rate for immunocompetent patients was 84.6%, versus 62.5% for immunocompromised patients. Data on immune status were unavailable for nine cases and were therefore excluded from this comparison. The most common comorbidity was prematurity. Out of 19 preterm neonates 10 survived (52.6%) and 9 died. Survival for full-term infants and older children was 69.8% (30 out of 43 cases). None of the included studies reported adverse effects or toxicity associated with antifungal therapy.

### 3.5. Antifungal Resistance

Minimum inhibitory concentration (MIC) values were considered to assess antifungal susceptibility among *C. auris* isolates. Currently, no species-specific clinical breakpoints have been established, although tentative interpretive criteria have been proposed by EUCAST and the CDC [30,31]. The correlation between microbiological MIC thresholds and clinical outcomes remains uncertain [12]. Early *C. auris* strains demonstrated low fluconazole susceptibility (MIC ≈ 4 mg/L, determined in-house by EUCAST), and isolates with similarly low azole MICs continue to be reported, particularly from South America. However, most *C. auris* isolates exhibit fluconazole MIC values >16 mg/L and possess acquired resistance mechanisms. Because of the scarcity of true wild-type, non-outbreak isolates, a reliable epidemiological cutoff value (ECOFF) for fluconazole cannot currently be defined. While the use of fluconazole in cases with lower MICs (≤4 mg/L) has been proposed as an amphotericin B alternative, EUCAST presently considers the available data insufficient to recommend fluconazole therapy for *C. auris*, even when MIC values appear low [30].

Antifungal susceptibility was summarized according to the CDC’s tentative breakpoints. Calvo et al. reported that all isolates were resistant to fluconazole but provided only aggregate MIC data for other antifungal agents; therefore, only fluconazole results from that study were included [22]. Chowdhary et al. also did not report individual MIC values and were excluded from drug-specific analyses [23]. Among the 53 isolates for which data were available, 8 (15%) were susceptible to fluconazole and 45 (85%) were resistant. Amphotericin B susceptibility was documented in 17 isolates, with 8 (47%) susceptible and 9 (53%) resistant. All isolates tested against other antifungal agents, including itraconazole, voriconazole, micafungin, caspofungin, anidulafungin, posaconazole, and isavuconazole, were reported as susceptible. No instance of pan-resistance was identified among the included studies.

### 3.6. Risk of Bias Assessment

The risk of bias was appraised using the relevant JBI Critical Appraisal Checklists. Ten studies were rated as low risk, and four as moderate (Table 4) [15]. The main methodological weaknesses included incomplete demographic or outcome data, and incomplete or non-consecutive inclusion of participants in case series.

## 4. Discussion

### 4.1. Empirical Treatment and Effective Identification

The treatment patterns observed in the eligible studies reveal that empiric therapy was mainly fluconazole-based, largely driven by early misidentification of *C. auris* as other *Candida* species, protocols of prophylactic treatment, especially for outbreaks or patients with severe comorbidities, or reliance on empirical *Candida* coverage. Despite this, survival among empirically treated patients (64%) was almost identical to that of patients receiving targeted therapy (65.3% for echinocandins and 70.6 for amphotericin B formulations). This suggests that early fluconazole administration did not worsen outcomes meaningfully in the cases above, possibly because patients underwent rapid pharmacotherapy adjustments, transitioning to echinocandins or amphotericin B once *C. auris* was identified. The critical factor determining the therapeutic outcomes does not appear to be the initial empirical drug of choice, but rather the timeliness of correction toward a suitable agent once the pathogen is successfully recognized. Once again, the necessity of robust and reliable identification methods is underlined.

Traditional biochemical as well as phenotypic assays, like API 20C, Vitek 2, Phoenix, and MicroScan, frequently misidentify *C. auris* as other *Candida* species, or even *Rhodotorula* spp., leading to inappropriate drug choices and crucial delays in infection management [32]. Misidentification rates exceeding 40% have been reported in European quality control trials [33]. MALDI-TOF mass spectrometry has substantially improved diagnostic accuracy but remains culture-dependent and requires validated *C. auris* spectra [34]. It may also struggle to distinguish between geographically distinct clades. Identification methods targeting the internal transcribed spacer (ITS) and D1/D2 regions of large subunit rRNA provide a culture-independent, highly specific alternative for *C. auris* identification, currently regarded as the diagnostic gold standard. Beyond species-level identification, molecular typing enables clade differentiation, offering valuable epidemiological insights, a helpful asset in outbreak management [35].

### 4.2. Targeted Therapy

Among targeted regimens, echinocandins and amphotericin B formulations were used with similar frequency, with similar survival outcomes between the two groups of patients. The slightly higher survival of patients within the amphotericin B group (70.6% vs. 65.3%) should be interpreted with caution, given the small sample size and the fact that amphotericin B was selected for neonates and cases with CNS or urinary tract infections, signifying a different clinical profile. Nevertheless, the absence of meaningful survival differences between the two drug classes in targeted therapy suggests that both agent categories remain viable options for targeted *C. auris* treatment once the pathogen is correctly identified. Choice should be driven by infection site and patient age, with echinocandins displaying strong anti-biofilm activity, an advantageous property for combatting infections related to CVCs or other colonized devices [36].

### 4.3. Combination Therapy and Effectiveness

Although combination therapy is not routinely recommended, several clinical reports, including those by Ozen et al. and Alvarado et al., have documented apparent synergistic effects between azoles and echinocandins, leading to improved microbiological clearance in severe or refractory cases [16,26,37]. A minority of patients (n = 6) received combination therapy in this review, having higher apparent survival (83%) compared to monotherapy (67%). Similarly, sequential monotherapy showed 80% survival, with this approach frequently used as a response to clinical deterioration, possible antifungal resistance, or failure of the initial pharmacotherapy. Interestingly, the survival observed in the “multiple agents, uncertain if combination therapy” category is lower than all the above (54%). This likely reflects the clinical complexity of these cases, since many among them were documented as part of high-risk nosocomial outbreak reports. The above findings further support the notion that treatment strategy was strongly shaped by case severity and clinical instability, rather than standardized therapeutic planning. While more aggressive or multidrug regimens were not inherently more effective in the cases reviewed, but rather were indicative of more complicated clinical courses, the observed pattern aligns with the prior literature supporting combination antifungal therapy for persistent or multidrug-resistant fungal infections [37,38,39]. As a result, further exploration in *C. auris* cases is warranted.

### 4.4. Risk Factors and Prognosis

While the pathogenic attributes of *C. auris* have yet to be completely understood, host factors clearly influenced outcome. Immunocompromised patients experienced substantially lower survival (60%) than immunocompetent children (60%), highlighting the importance of immune status in recovery from invasive *C. auris* infection. Prematurity also emerged as a major adverse prognostic factor, with preterm neonates exhibiting the lowest survival (53%), consistent with their vulnerability to sepsis, poor barrier defenses, and limited ability to mount effective immune responses. The above trends underline that mortality in pediatric *C. auris* patients is strongly driven by comorbidities and vulnerabilities rather than antifungal selection alone. None of the included studies reported antifungal toxicity, which may reflect true safety in pediatric use or incompleteness in side-effect reporting. Nonetheless, the absence of documented adverse effects suggests that antifungal-related harm was not a major contributor to mortality in the cases included in the present review.

### 4.5. Antifungal Resistance and Patterns

The susceptibility data demonstrate that fluconazole resistance was extremely common (85% of cases), explaining its limited effectiveness once the pathogen was identified. In contrast, other azoles showed potentially significant activity in some cases. Amphotericin B resistance was also observed (53%), but clinical survival remained acceptable in patients treated with amphotericin B formulations, suggesting that MIC values may not fully predict clinical outcomes in this pediatric population. The universal susceptibility observed for echinocandins in reported isolates aligns with their status as the current cornerstone of *C. auris* pharmacotherapy [12]. No instances of pan-resistance were identified within the included studies, yet the use of antifungals combination or sequential antifungals in numerous cases may reflect clinician concern for emerging resistance [40]. Overall, the combined clinical and microbiological findings emphasize that early recognition, rapid adjustment to active treatment, and careful attention to host vulnerability remain the key determinants of clinical outcome in pediatric *C. auris* infections.

### 4.6. Limitations and Future Investigations

The present review highlights significant gaps in the current understanding of *C. auris* infections in pediatric populations. The available evidence is derived predominantly from case reports, small case series, and nosocomial outbreak investigations, often featuring incomplete demographics, details on treatment regimens, and reported clinical outcomes. Such heterogeneity hinders the establishment of firm associations between antifungal therapy and patient prognosis, especially to compare outcomes expected from different antifungal classes. The inability to assess attributable or time-defined mortality related to *C. auris* infection was also a major limitation. Most studies reported only crude or overall mortality without specifying whether death was directly attributable to invasive *C. auris* infection. Only two fatal cases explicitly addressed infection attribution to mortality, and fewer than one-third of reported deaths included information on 30-day survival. As a result, overall mortality in pediatric *C. auris* infection should be interpreted as a composite marker, reflecting the severity of an underlying disease, or other comorbidities, rather than infection-specific lethality.

Furthermore, antifungal susceptibility testing was performed inconsistently, with different methodologies and, in many cases, unclear interpretive breakpoints, complicating case-by-case comparisons between different studies. Until recently, the absence of standardized *C. auris*-specific breakpoints further obscured the clinical relevance of in vitro data, while incomplete or aggregate MIC reporting prevents a concrete assessment of expected drug performance [41]. The limited pharmacokinetic and pharmacodynamic data for antifungal agents in neonates and children is equally important, restricting evidence-based dosing recommendations. Therapeutic decisions in this demographic are usually extrapolated from adult studies, despite substantial physiological differences in drug metabolism and tissue diffusion. Moreover, the use of empirical treatment in the absence of definitive pathogen identification raises concern for suboptimal treatment and the potential of antifungal resistance development [42]. The extensive fluconazole resistance observed among isolates underscores the need for targeted antifungal stewardship initiatives and increase in laboratory capacity for routine susceptibility testing in resource-limited settings [43].

Future research should prioritize prospective clarifying treatment strategies and establish a connection with clinical outcomes in pediatric *C. auris* infections. Establishing a centralized registry of pediatric cases, including detailed clinical, pharmacological, and microbiological data, would enhance global surveillance and improve therapeutic decision-making. The exploration of combination regimens to combat the development of antifungal resistance is also warranted to inform clinical practice, alongside the validation of pediatric-specific dosing models. Finally, the integration of molecular diagnostics, such as ITS sequencing, into routine workflows will be immensely helpful for accurate species and geographic clade identification, as well as resistant strain detection. Addressing these gaps will be critical to mitigate the growing threat of *C. auris* in vulnerable pediatric populations, safeguarding the effectiveness of antifungal agents in the years to come.

## 5. Conclusions

*Candidozyma auris* has proven a growing global challenge in nosocomial settings, even under appropriate preventive or correctional measures. Considering the pathogen’s persistence in hospital settings and medical devices, alongside the high mortality rate associated with *C. auris* candidemia, the urgency of the matter becomes evident. The arsenal of antifungals is finite and shallow, emphasizing the pressing need to develop novel antifungals capable of overcoming multidrug and pan-resistant fungal strains. Fungal regulation of virulence, biofilm formation, and integrity and cellular stress responses may provide promising avenues for future research on the matter. Finally, the establishment and implementation of robust yet versatile antifungal stewardship programs promises to empower effective empirical antifungal therapy, taking into account local epidemiology, susceptibility trends, and precise diagnostics. Antifungal selection based on evidence, for empirical or targeted pharmacotherapy, should form the mainframe of stewardship frameworks, with therapeutic drug monitoring (TDM) optimizing dosing, route of administration, and duration of therapy, while preserving the effectiveness of current antifungals and achieving more favorable patient outcomes.

## Figures and Tables

**Table 1:** PICO table outlining population (P), intervention (I), comparator (C), and outcome (O).

Population	Pediatric patients, aged 0–18 years, with confirmed *Candida auris* infection
Intervention	Administration of antifungal treatment (echinocandins, azoles, amphotericin B, novel antifungals)
Comparator	Studies comparing different antifungal regimens In the absence of direct comparators, studies reporting outcomes for a single antifungal treatment will also be considered to ensure inclusion of all available pediatric evidence
Outcome	Clinical outcomes associated with each antifungal treatment

**Table 2:** Timing information and outcomes.

Therapy Category	Definition	Total Patients (N)	Agents Used	Patients Receiving Each Agent	Survival (n, %)
Empiric Therapy	Initiated before identification of *C. auris*	27	Fluconazole	25	16 (64%)
Targeted Therapy	Initiated after confirmation or susceptibility data	41	Echinocandins	26	17 (65.3%)
			Amphotericin B Formulations	17	12 (70.6%)
Timing Unclear	Timeline not described	17			

**Table 3:** Combination therapy and outcomes.

Therapy Category	Total Patients (n)	Survival (n, %)
Combination Therapy	6	5 (83%)
Monotherapy	25	17 (68%)
Sequential Monotherapies	5	4 (80%)
Multiple Agents, Unsure if Combination Therapy	26	14 (53.8%)

**Table 4:** Risk of bias results.

Study (Author, Year)	Design	JBI Checklist Used	Overall Risk of Bias
Ozen et al., 2025 [16]	Case Report	JBI Case Report	Low
Shin et al., 2025 [18]	Clinical Note	JBI Case Report	Low
Das et al., 2025 [17]	Case Report	JBI Case Report	Low
Abastabar et al., 2019 [19]	Case Report	JBI Case Report	Low
Anirima et al., 2024 [20]	Case Series	JBI Case Series	Moderate
Lee et al., 2011 [21]	Case Series	JBI Case Series	Low
Calvo et al., 2016 [22]	Outbreak Report	JBI Case Series	Low
Chowdhary et al., 2020 [23]	Case Series	JBI Case Series	Moderate
Chandramati et al., 2020 [24]	Outbreak Report	JBI Case Series	Moderate
Ramya et al., 2021 [25]	Case Series	JBI Case Series	Low
Alvarado et al., 2021 [26]	Case Series	JBI Case Series	Low
Mirhendi et al., 2022 [27]	Case Report	JBI Case Report	Low
Erkol et al., 2025 [28]	Case Report	JBI Case Report	Low
Chen et al., 2018 [29]	Case Series	JBI Case Series	Moderate

## Data Availability

The original data presented in the study are openly available in Open Science Framework (OSF) at https://doi.org/10.17605/OSF.IO/F5KBZ.

## Supplementary Materials

The following supporting information can be downloaded at https://www.mdpi.com/article/10.3390/microorganisms14010228/s1. Table S1. PRISMA 2020 Checklist. Table S2: The complete search strategy for PubMed (October 2025).

## Abbreviations

The following abbreviations are used in this manuscript:

**Abbreviation**	**Full Form/Explanation**
MIC	Minimum Inhibitory Concentration
CVC	Central Venous Catheter
ITS	Internal Transcribed Spacer
MALDI-TOF MS	Matrix-Assisted Laser Desorption Ionization–Time of Flight Mass Spectrometry
PICU	Pediatric Intensive Care Unit
ICU	Intensive Care Unit
CT	Computed Tomography
LP	Lumbar Puncture
NICU	Neonatal Intensive Care Unit
EUCAST	European Committee on Antimicrobial Susceptibility Testing
CDC	Centers for Disease Control and Prevention
ECOFF	Epidemiological Cutoff Value
PRISMA	Preferred Reporting Items for Systematic Reviews and Meta-Analyses
OSF	Open Science Framework
PICO	Population, Intervention, Comparator, Outcome
JBI	Joanna Briggs Institute
IQR	Interquartile Range
TDM	Therapeutic Drug Monitoring
RDS	Respiratory Distress Syndrome
UTI	Urinary Tract Infection
CNS	Central Nervous System
SFC1	Surface Colonization Factor 1
